# Granular osteoconductive biopolymer and predictive analysis of its physical property changes in maxillofacial surgery

**DOI:** 10.1016/j.csbj.2025.06.021

**Published:** 2025-06-06

**Authors:** Denis G. Alekseev, Oleg V. Slesarev, Darya V. Malchikova, Vyacheslav G. Belanov, Natalia A. Maksimenko, Vladimir I. Platonov, Ravshanbek B. Abdullaev

**Affiliations:** a Samara State Medical University, Samara 443079, Russia; b Samara National Research University, Samara 443079, Russia; c Tashkent Medical Academy, Tashkent 100109, Uzbekistan

**Keywords:** Innovative materials, Biotechnology, Biopolymers, Physical property, Tissue engineering, Maxillofacial surgery

## Abstract

Dry bulk fractions of osteoconductive granular biopolymers (OGB) are a common choice for addressing jawbone defects. These, in conjunction with an insulating biological membrane, form bioengineered scaffold structures. In the recipient site, the OGB fraction undergoes biotransformation, morphing from a bulk granular fraction into a stable conglomerate. The biotransformation of the OGB fraction is a physical process initiated by the biological environment of the recipient site. This process is characterized by granule compaction due to reduced intergranular space volume, leading to an uncontrolled decrease in the planned volume of the OGB. This is one of the causes of postoperative complications.

This study aims to identify prognostic indicators that characterize the dynamics of changes in the physical property of the total volume of the OGB fraction following the addressing of a jawbone defect (in the postoperative period). To minimize these limitations, the study substantiated the use of prognostic indicators that can characterize the behavior of the entire volume of the OGB fraction within the recipient site during the postoperative period. The study presents the true adsorption capacity (AC_T_) and compaction coefficient (C_C_) of the OGB fraction. Gas adsorption, Fourier transform infrared spectroscopy, and scanning electron microscopy substantiate that preoperative preparation methods enhance the physical property of the OGB fraction. These methods include the extraction of coarse and fine industrial dust from the granule surfaces and the removal of air pockets from the granule pores.

Thus, studying OGB behavior in the recipient site justifies the use of prognostic indicators, which is clinically significant. These indicators enable the surgeon to design a 3D sculpture of the bioengineered scaffold structures, considering the changes in the physical property of the OGB fraction during the postoperative period. This approach minimizes the risks of postoperative complications caused by uncontrolled compaction of the OGB fraction. Preoperative preparation of the OGB fraction can significantly enhance its adsorption capacity, increasing it by up to 25 %.

## Graphical abstract

**Figure:**
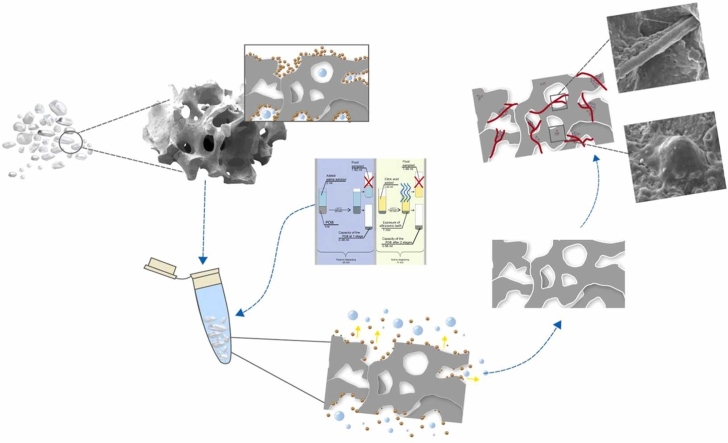

## 1 Introduction

The surgical reconstruction of geometrically complex-shaped jawbone defects relies on meticulously engineered scaffold systems. These three-dimensional (3D) architectures must balance clinical functionality with precise anatomical adaptation, maintaining both defect conformity and volumetric stability throughout the healing process. Typically constructed from desiccated osteoconductive granular biopolymer (OGB) mixtures, these scaffolds fulfill three essential requirements: controlled biodegradation, structural integrity, and osteoconductive capacity [1]. Their long-term performance depends on the interaction between the OGB’s physicochemical properties and the host environment [2], [3].

Within the implantation site, OGB particles undergo a remarkable biological metamorphosis. While their internal matrices gradually remodel into native bone tissue, their external surfaces function as dynamic extracellular platforms providing adhesion sites for migrating cells, supporting proliferation, and enabling tissue colonization [4], [5], [6]. This osteoconductive program unfolds through precisely orchestrated biophysical cues, where the OGB's material properties directly modulate signal transmission to surrounding cellular environments [7].

Clinically utilized OGB formulations exhibit heterogeneous granulometry, displaying characteristic bulk material properties including particle morphology and size distribution, pore architecture (volume, geometry, connectivity) and surface topography and crystallinity [8], [9], [10], [11]. Unlike monolithic implants, granular OGB systems offer next clinical advantages:

• Adaptive filling of irregular defect geometries [12], [13];
• Extensive surface area (internal/external) for biological interaction;
• Interparticle spaces facilitating vascular ingrowth and molecular transport [14], [15], [16], [17].

As demonstrated by Stuckensen et al., biomimetic frameworks can intrinsically guide cellular behavior through structural and biochemical cues, promoting autonomous matrix deposition [18]. This process relies on sophisticated cell-matrix communication mediated by trans-interaction mechanisms and heterophilic binding dynamics [19]. The scaffold's drainage capacity and adsorption characteristics critically influence molecular trafficking patterns. Modern fabrication techniques enable precise tuning of porosity gradients, surface roughness profiles and mechanical property distribution [20], [21].

Notably, surface topography directly modulates hydrodynamic behavior-increased roughness correlates with reduced permeability and fluid shear stress [21], enabling controlled molecular drift through intergranular spaces [22]. The platelet-fibrin matrix provides essential physical stabilization through progressive granule approximation, interparticle space reduction, and final conglomerate formation [23]. Future directions of key researches in granular scaffold optimization include:

• conglomeration kinetics control – regulating drainage system development;
• transport capacity enhancement – optimizing molecular exchange while reducing hypoxia;
• surface engineering – refining granule/pore morphology during production;
• clinical preparation protocols – standardizing pre-implantation processing.

Emerging solutions will likely combine advanced materials, novel processing techniques and hybrid cellular approaches [24].

When planning a surgery for the bone defect addressing with OGB, it is important to base the process on prognostic indicators that characterize the dynamics of changes in the physical property of the granular fraction in the recipient site. The identification of such indicators characterizing the transformation is a promising direction for optimizing the production and clinical use of OGB. Developing of a toolkit that includes a set of prognostic indicators that characterize changes in the physical property of the OGB in the recipient site is highly demand. Surgical success requires prognostic modeling of OGB's in vivo transformation using key parameters:

• adsorption capacity;
• natural compaction coefficient;
• pore surface area metrics;
• through-pore ratio analysis.

Developing this predictive toolkit represents a critical step toward optimized OGB applications in regenerative dentistry. The use of prognostic indicators will allow optimal planning of the surgical protocol, stabilize the volume of the scaffold at the recipient site, and control the dynamics of the biotransformation of OGB in the postoperative period. Thus, this study is focused on developing tools for prognostic analysis of the OGB granular fraction transformation in recipient site.

## 2 Materials and methods

To achieve the objective, the study examined the physical property of the OGB that determine the stability of the volume and biotransformation dynamics of the scaffold in the postoperative period. The investigation included six officinal OGBs in granular fraction:

• Maxresorb® 0.5–1 mm – synthetic biphasic calcium phosphate granules (Botiss biomaterials GmbH, Germany);
• Bio-Oss® 0.25–1 mm – granules of mineral portion of natural bovine bone (Geistlich, Switzerland);
• Cerabone® 0.5–1 mm – granules of mineral portion of natural bovine bone (Botiss biomaterials GmbH, Germany);
• Xenograft Collagen® 0.25–1 mm – granules of decellularized and freeze-dried xenogeneic cattle bone (BioOST, Russia);
• Osteon II® 0.2–0.5 mm – synthetic biphasic calcium phosphate supplemented with composition of 30 % hydroxyappatite and 70 % β-tricalcium phosphate (Genoss, South Korea);
• Mineralized spongy powder (MSP) 0.5–1 mm – granules of decellularized and freeze-dried allogeneic bone (LYOPLAST-С®, Russia).

### 2.1 Calibration of the research protocol

A fraction of natural limestone (GLF) of biogenic origin with a granulometric composition of 0.5–1 mm was used to calibrate the study protocol.

#### 2.1.1 Production of a granular limestone fraction

The limestone was crushed under pressure and in a mill to obtain a granular fraction.

Granulometric calibration of the fractioned granules: granular fraction of limestone was loaded into a sieve with a mesh size of 2 mm and sifted on a sieve shaker S-30 (VIBROTEHNIK, Russia) for 1 min. The vibration amplitude of the shaker sieves was 0.5 mm, and the vibration frequency – 1500 vibrations per minute.

The fraction of granules with 2 mm of size was placed in a sieve with a mesh size of up to 1 mm and sieved under similar conditions.

To obtain 0.5 mm granules, a 1 mm limestone fraction underwent sieving through a 0.5 mm mesh under similar conditions. Subsequent sieving through a 0.4 mm mesh removed the residual fraction.

The limestone fraction with a granulometric composition of 1 mm was mixed with an equal amount of the granule fraction with a 0.5 mm of size.

The resulting limestone fraction (GLF), with a granulometric composition of 0.5–1 mm, underwent fine dust removal using a low stream of dry air.

#### 2.1.2 The method of degassing and dust extraction

The measurement of the GLF fraction on analytical scales yielded a dry sample mass (m_1_ = 2 g). The elimination of coarse and fine particulate matter, as well as air bubbles were removed from the OGB fraction via degassing and dust extraction [25]. The method of degassing and dust extraction consisted of two consecutive stages.

The first stage was passive degassing and dust extraction with removal of coarse particulate matter and air from surfaces and voids. The first stage proceeded in saline solution (SS), since its osmotic pressure (0.9 %) corresponds to the osmotic pressure of blood plasma. In the thermostat, the SS temperature was adjusted to 37ºC, corresponding to the recipient site's temperature. GLF was placed into the test tube of the thermostat and filled with SS with a ratio of 1:2 per 1 cm of GLF. The procedure then included an additional 2 mL of saline solution. Twenty minutes later, the GLF was separated from the SS using a pipette dispenser – a mechanical single-channel dispenser with a variable volume of 20–200 µl.

The second stage was active degassing and dust extraction with removing of fine particulate matter and residual gases from the mouths of interstitial channels and voids. According to [26], [27], the GLF was filled with a citric acid (pH 1) solution at 37°C for 10 min. For this, a saturated acid solution was prepared by slowly adding of anhydrous citric acid to 50 mL of distilled water and using of a magnetic stirrer Microfix (JP Selecta S.A., Spain) to stir the solution. The process continued with the addition of citric acid crystals until reaching pH 1 (determined using a pH meter).

After ten minutes of GLF being in citric acid solution, ultrasound (18 kHz, 250 W, exposure 60 s) performed active degassing and dust extraction in an ultrasonic bath UZU-0.25 (UPPO, Russia). After ultrasound, citric acid was separated from the GLF using a pipette dispenser. To remove acid residues, the GLF underwent immersion in a SS at 37ºC and treating in an ultrasonic bath (18 kHz, 250 W, exposure 60 s).

All six officinal OGBs underwent identical preparation following the sequence of operations described in this paragraph.

### 2.2 Determination of the biocompatibility of OGB preparation

The study proceeded in vitro on 36 samples – 18 in each of the next two groups (accordingly, 3 repeats) with control:

• Group 1 – samples of all six OGBs without preparation according to the protocol described in paragraph 2.1.2.
• Group 2 – samples of six OGBs prepared according to the protocol described in paragraph 2.1.2.
• Group 3 – the control group with the mesenchymal stem cells (MSCs) in culture medium, without samples of OGBs.

MSCs obtained from the human umbilical cord (donor No. 7000400025628, cultures of the second passage) served to evaluate the cytotoxicity of the developed protocol of the degassing and dust extracting method. The viability of the cells during freezing was 96 %. Before the experiment, MSCs was thawed in a water bath at 37°C for 2.5 min then washed twice from the cryoprotection in a laminar flow cabinet KS-12 Herasafe (Thermo **Fisher** Scientific, USA) with a sterile solution of phosphate-salt buffer (BioloT Ltd, Russia). The viability of MSCs before the experiment was 94 % amounting to 5.8 million.

The experimental protocol positioned samples from each of three group in 100 cm culture Petri dishes (SPL, Korea) with sterile alpha-MEM liquid growth medium and alanine-glutamine (BioloT Ltd, Russia). In groups No. 1 and 2, MSCs of 400,000 were transferred to the samples’ surface using a variable volume dispenser. In group No. 3 the same number of MSCs was used. 15 min after the cell suspension transferring, the experiment included 2 mM of L-glutamine (BioloT Ltd, Russia) to each dish. Then dishes were placed into the CO_2_ incubator CB 210 (Binder, Germany) and incubated under standard conditions for 8 days. Daily visual inspections monitored the culture, with concurrent cell morphology evaluations. On the eighth day trypsin-EDTA solution detached cells from culture dishes (PanEco, Russia) with following cells disaggregation from the plastic according to the protocol. The protocol includes microscopic control every 5 min of trypsinization.

The Countess II FL Automated Cell Counter (Thermo Fisher Scientific, USA) was used to assess the viability of MSCs. The study measured both the proliferative index and doubling rate of MSC cultures. The morphology of the cultured MSCs was evaluated dynamically at all stages of cultivation using an AXIO Observer A1 microscope (Carl Zeiss, Germany).

### 2.3 Determining the true adsorption capacity indicator of the OGB

The determining of the OGB granular fraction’s apparent adsorption capacity (AC_A_) implies the use of the following physical indicators and characteristic: water absorption by mass of the OGB samples, including intergranular spaces (W_mА_), water absorption by mass of the OGB samples, excluding intergranular spaces (W_mB_), the volume of micropores, the presence of macropores and through pores in the granules.

#### 2.3.1 Determining the apparent adsorption capacity (AC_A_) of the OGB

The volume (V_1_) of the extracted SS was determined at the end of the first degassing and dust extraction stage according to the protocol described in paragraph 2.1.2. using a pipette dispenser (a mechanical single-channel dispenser with a variable volume of 20–200 µl) in an Eppendorf (volume of 2 mL) (Table 1).

**Table 1:** Physical indicators for calculating the true adsorption capacity (AC_T_) of the OGB samples.

**Indicator**	**Formula**
Apparent adsorption capacity of the OGB (АC_A_), mL	АC_A_ = (V_3_ - V_1_) + (V_1_ - V_2_)
Water absorption by mass, including intergranular spaces (W_mА_), %	W_mА_ = [(m_2_ - m_1_) / m_1_] × 100 %
Water absorption by mass, excluding intergranular spaces (W_mB_), %	W_mB_ = [(m_3_ - m_1_) / m_1_] × 100 %

*V_1_,V_2_,V_3_ (mL); m_1_, m_2_, m_3_ (g).

To determine the volume (V_2_) according to the protocol described in paragraph 2.1.2. at the stage of active degassing and dust extraction, the OGB samples was filled with the citric acid solution in the volume (V_1_). At the end of the second stage, the volume (V_2_) of the extracted liquid was determined using a pipette dispenser in an Eppendorf (volume of 2 mL).

The volume (V_3_) is the initial volume of liquid used in the first stage of degassing and dust extraction (mL).

The apparent adsorption capacity of the OGB was determined by the following formula: АC_A_ = (V_3_-V_1_) + (V_1_-V_2_)

#### 2.3.2 Determining the indicator of the water absorption by mass of the OGB, including intergranular spaces

• The mass of the dry OGB samples was measured on analytical scales: m_1_.
• The mass of the OGB samples measured after exposure to two stages of degassing according to the protocol of paragraph 2.1.2.: m_2_.
• The indicator of the water absorption by mass of the OGB samples, including intergranular spaces (W_mА_), was determined according to the following formula: W_mA_ = [(m_2_ - m_1_) / m_1_] × 100 % (Table 1).

#### 2.3.3 Determining the indicator of the water absorption by mass of the OGB, excluding intergranular spaces

To remove the residual liquid from the intergranular spaces, the OGB samples sat on a slide while movement along the glass surface continued until the liquid leaked out of the sample so the mass (m_3_) of the OGB samples was determined.

The indicator of the water absorption by mass of the OGB samples, excluding intergranular spaces (W_mB_), was determined according to the following formula: W_mB_ = [(m_3_ - m_1_) / m_1_] × 100 %.

#### 2.3.4 Scanning electron microscopy

Fragments of the OGB samples were mounted on a double-coated, carbon-conductive tape and then sputter-coated with gold using universal vacuum magnetron sputtering device UNICOAT 600 t (Elan-Praktik, Russia). A Tescan Vega (TES-CAN, Czech Republic) equipped with an Inga Energy SEM microanalyzer (Oxford Instruments pic, UK) conducted topographic analysis of the samples' surface in secondary electron detection mode at 30 kV accelerating voltage. This way the study figured out the area of the outer-inner surfaces of channels and pores, the volume of the pores and channels, and the ratio of pores to channels in the OGB. The working distance was adjusted to obtain a suitable magnification.

#### 2.3.5 Gas adsorption

Gas adsorption let us to determine the specific surface area of the OGB samples. The textural characteristics of the samples were measured on a Quantachrome Autosorb-1 (Quantachrome Instruments, USA) adsorption porosimeter by low-temperature nitrogen adsorption. To characterize the specific surface area, the Brunauer-Emmett-Teller (BET) model was used at a relative partial pressure P/P0 of 0.05–0.3. The total pore volume and pore size distribution were calculated from the desorption curve using the Barrett-Joyner-Halenda (BJH) model. Before testing, the OGB samples were calcinated at a temperature of about 300°C to remove water adsorbed on the surface.

#### 2.3.6 Calculation of the true adsorption capacity indicator of the OGB

The method of AC_T_ calculating was determined by the nature of the distribution of macropores and micropores in OGB samples’ granules:

• For such OGB samples as Maxresorb, Cerabone, Osteon Ⅱ, and MSP, AC_T_ corresponds to AC_A_.
• For such OGB samples as Xenograft Collagen, АC_T_ = АC_A_ × ((W_mА_ - W_mB_) + W_mB_ × 0,85) / W_mА_.
• For such OGB samples as Bio-OSS, АC_T_ = АC_A_ × (W_mА_ - W_mB_) / W_mА_.

### 2.4 Determining the compaction coefficient of the OGB

The procedure included pouring the OGB samples into a test tube without compaction, and the height of the column was marked as h_1_. The next step involved mounting the test tube on a vibrating table VD 30 vibration drive (VIBROTECHNIK, Russia) and compacted content with an oscillation amplitude of 0.25 mm for 10 min. The height of the column of the OGB samples obtained after compaction was marked as h_2_. The compaction coefficient (С_С_) of the OGB samples calculating according to Table 2.

**Table 2:** Determining the compaction coefficient (С_С_) of the OGB samples.

**Indicator**	**Formula**
Real volume of the OGB sample in its natural form	V_E_ = (π × r^2^ × h_1_) / 4
Average density	ρ_е_ = m_1_ / V_E_
Water adsorption of the OGB sample by mass	W_mЕ_ = [(m_4_ - m_1_) / m_1_] × 100 %
Water adsorption of the OGB sample by volume	W_vЕ_ = [(m_4_ - m_1_) / V_E_] × 100 %
Dependency coefficient	d = W_vE_ / W_mE_
The volume of the OGB sample after compaction	V_C_ = (π × r^2^ × h_2_) / 4
Water adsorption of the OGB sample by mass after degassing	W_mC_ = [(m_5_ - m_1_) / m_1_] × 100 %
Water adsorption of the OGB sample by volume after degassing	W_vC_ = [(m_5_ - m_1_) / V_C_] × 100 %
Dependency coefficient after compaction	d_max_ = W_vC_ / W_mC_
Density of the OGB sample after compaction	ρ_c_ = m_1_ / V_C_
**The compaction coefficient of the OGB sample**	C_C_= d_max_ / d

*r - radius of the container (cm); $h_{1}$- the height of the OGB fraction in its natural form (cm); $h_{2}$- the height of the OGB sample after compaction (cm); $m_{1}$- the mass of the OGB fraction in the dry state (g); $m_{4}$- the mass of the OGB sample saturated with SS (g); $m_{5}$- the mass of the OGB sample after the conducted degassing stages (g).

### 2.5 Fourier transform infrared spectroscopy

Fourier transform infrared spectroscopy (FTIR) assessed the chemical composition and major functional groups present in the samples. Before analysis, a mortar and pestle homogenized all six OGB samples under dry conditions. Then the spectra were recorded in the 400–4000 cm^−1^ using a spectrometer Nicolet IS-50 (Thermo Fisher Scientific, USA) in attenuated total reflection mode. The spectrometer recorded each spectrum at room temperature with a resolution of 6 cm^−1^, with the number of scanned samples equal 32.

### 2.6 Nonlinear regression fitting for evaluation of the degassing rate

To assess the rate of degassing, in the study used triplicate experiments with the interval from 0 min (start of degassing) to 20 min (end of degassing). The volume of the liquid was recorded every 2.5 min. The software used for statistical analysis is Statistical Package for the Social Sciences SPSS® 25.0 (IBM®, USA).

The arithmetic means of the remaining liquid volume, the standard deviation, the mean error, and the 95 % confidence interval boundaries were calculated at each studied time point.

The experimental data 9-time points with 3 measurements in each, for a total of 27 pairs of observations are smoothed with a high degree of accuracy by a 4-parameter logistic curve, the equation of which in general form is as follows:

f (t) = d + (c – d) / (1 + exp (– a × (t – b)),

where:

f (t) – the percentage of the initial volume of the liquid,

t – time in minutes,

d – the maximum percentage (without degassing); the study assumed this value to be 100 %,

с – parameter reflecting the minimum percentage of the initial liquid volume after the completion of degassing,

a – parameter reflecting the rate of degassing,

b – parameter reflecting the time of the semi-degassing.

## 3 Results

### 3.1 Biocompatibility of OGB preparation

The results of study of OGBs preparation (according to the developed protocol described in paragraph 2.1.2) on MSCs viability in 2 group, in comparison with non-prepared OGBs (group 1) and cells in culture medium (group 3) are shown in the Table 3.

**Table 3:** Indicators of the MSCs viability (M±SD).

OGB	Group number	Number of seeded cells in 1 mL *10^6^	Viability, %	Quantity collected cells in 1 mL *10^6^	Speed doubling in a day
	1	0.4	94.3	3.44 ± 0.29	0.4884 ± 0.2457
Cerabone	2	0.4	94.6	3.51 ± 0.38	0.5162 ± 0.1148
	3	0.4	96.0	3.62 ± 0.24	0.5212 ± 0.2354
	1	0.4	94.2	3.41 ± 0.27	0.4771 ± 0.2446
Xenograft	2	0.4	94.4	3.49 ± 0.36	0.5122 ± 0.1116
Collagen	3	0.4	96.0	3.61 ± 0.22	0.5221 ± 0.2349
	1	0.4	94.4	3.46 ± 0.26	0.4902 ± 0.2426
Osteon II	2	0.4	94.6	3.53 ± 0.31	0.5187 ± 0.1159
	3	0.4	96.0	3.64 ± 0.27	0.5218 ± 0.2351
	1	0.4	94.1	3.39 ± 0.30	0.4756 ± 0.2437
Maxresorb	2	0.4	94.5	3.49 ± 0.39	0.5094 ± 0.1109
	3	0.4	96.0	3.59 ± 0.23	0.5198 ± 0.2344
	1	0.4	94.7	3.48 ± 0.32	0.4971 ± 0.2429
Bio-OSS	2	0.4	94.9	3.56 ± 0.41	0.5112 ± 0.121
	3	0.4	96.0	3.61 ± 0.26	0.5229 ± 0.2347
	1	0.4	94.8	3.50 ± 0.33	0.4925 ± 0.2435
MSP	2	0.4	95.1	3.59 ± 0.41	0.5218 ± 0.1168
	3	0.4	96.0	3.64 ± 0.25	0.5247 ± 0.2361

**Fig. 1:**
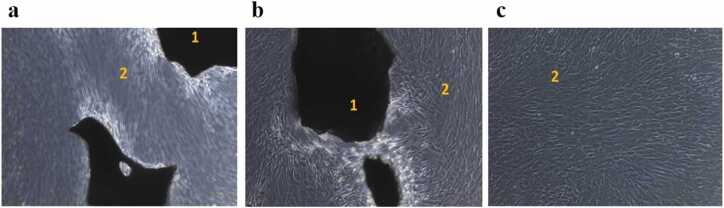
(**a**) The OGB (MSP) without preparation, group 1; (**b**) The OGB (MSP), prepared according to the protocol described in paragraph 2.1.2, group 2; (**c**) Control, cultured human MSCs without OGB (group 3). Digits: 1 – OGB (MSP); 2 – human MSCs.

Throughout the cultivation, MSCs had a typical spindle-shaped morphology, the edges of the cytoplasm were smooth, without any signs of apoptosis. Microscopic examination in groups 1 and 2 showed marginal attachment of MSCs to the surfaces of OGBs’ granules (Fig. 1**)**.

**Fig. 2:**
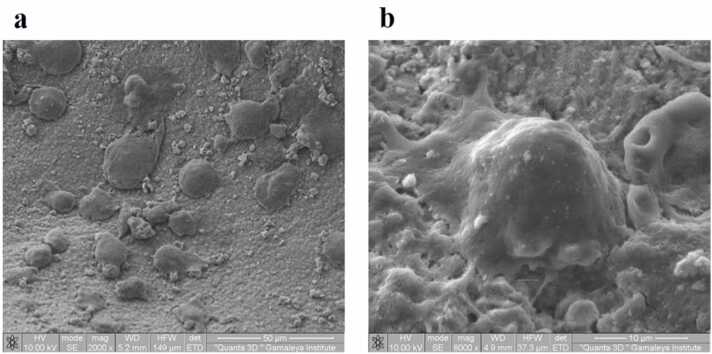
Scanning electron microscopy (SEM) of MSCs seeded on OGB (MSP). (**a**) Attachment, migration, and proliferation of MSCs on the surface of the OGB (MSP), magnification x2000; (**b**) MSCs 8 days after seeding on the OGB (MSP), magnification x8000.

Thus, after preparation of OGBs samples according to the protocol described in paragraph 2.1.2, the study did not reveal using scanning electron microscopy (SEM) any significant cytotoxic effect on human MSCs seeded on the OGBs granules (Fig. 2).

### 3.2 True adsorption capacity indicator of the OGB

#### 3.2.1 Apparent adsorption capacity of the OGB

**Fig. 3:**
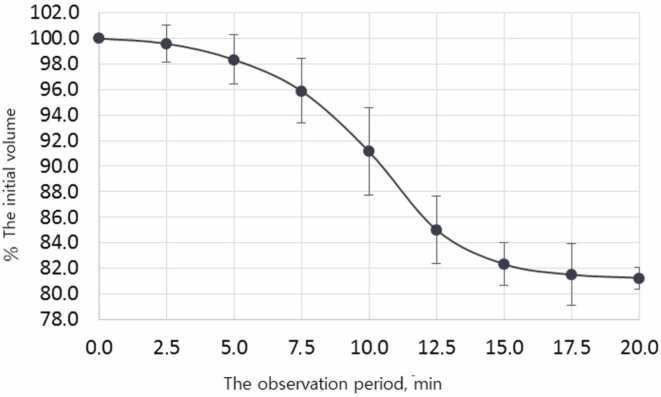
The process of degassing and dust extraction from the OGB sample (MSP). Error bars denote 95 % confidence intervals; the curve was fitted via nonlinear regression.

The study have established that at the stage of passive degassing, when the OGB samples are immersed for 20 min in a SS at 37ºC, there is a release of gas bubbles, and fine particulate matter. Fig. 3 shows that the initial descent of the curve is observed on 2.5 min after immersing of OGB sample in the SS, corresponding to a decrease in the initial volume of liquid and the release of gas bubbles.

The full release of gas bubbles occurs between 7.5 and 12.5 min. Between 13 and 17 min, fine particulate matter releases and fills the entire free volume of the solution as a suspension, partially settling afterward. From 17.5–20 min, the system reaches a plateau with no gas bubbling. Results showed initial liquid volume changes during degassing and dust extraction, along with average values, and corresponding 95 % confidence intervals (Table 4).

**Table 4:** Percentage of the initial fluid volume depending on time: average values and their 95 % confidence intervals.

Time, min	% of the initial volume, average values	% of the initial volume, lower bound 95 % confidence intervals	% of the initial volume, upper bound 95 % confidence intervals
0.0	100.0	100.0	100.0
2.5	99.6	98.2	101.0
5.0	98.3	96.4	100.3
7.50	95.9	93.3	98.4
10.0	91.2	87.7	94.6
12.5	85.0	82.4	87.7
15.0	82.3	80.7	84.0
17.5	81.5	79.1	83.9
20.0	81.3	80.4	82.1

In the SPSS statistical package environment, the observed values were approximated by an S-shaped function called 4-parameter logistic regression. Following resulting values of the regression coefficients and their standard errors reflect the parameters of the dependence of the percentage of degassing on time:

a = 0.51 ± 0.04
b = 10.10 ± 0.17
c = 81.01 ± 0.35

**Fig. 4:**
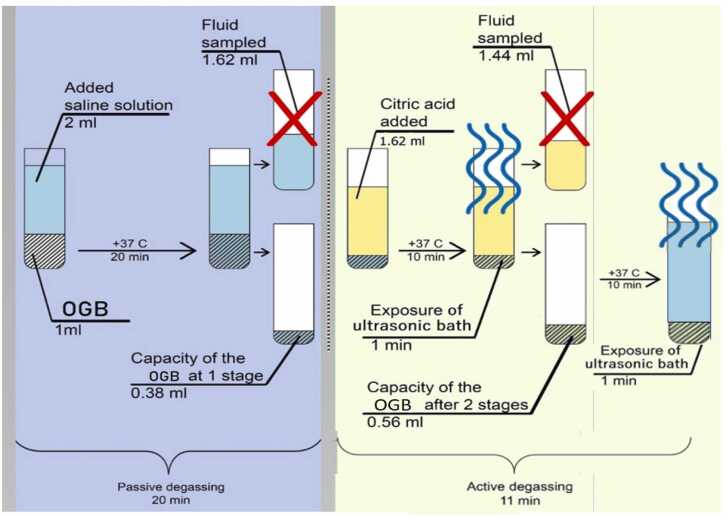
Changes in the volumes of liquids after degassing and dust extraction from the OGB fraction (Cerabone).

The equation for the dependence of the percentage of degassing on time t is as follows:

f (t) = d + (c – d) / (1 + exp (– a × (t – b)) = 100 + (81.01 – 100) / (1 + exp (– 0.51 × (t – 10.10)),

The coefficient of determination of the constructed model is R_2_ = 99 %, which characterizes it as very accurate. The developed mathematical model of the degassing process and dust extraction indicates that half of the gas present in the voids of the OGB fraction was released within 10 min. After 20 min from the beginning, the curve reached a plateau. 20 min after the passive degassing stage finishing, the measured volume of the extracted liquid amounted 1.62 mL. The difference between the initial fluid volume and that after passive degassing was 0.38 mL (2 mL – 1.62 mL = 0.38 mL). At the end of the active degassing and dust extraction stage, the residual volume was equal to 1.44 mL. The difference between the volumes of the liquid before and after active degassing was 0.18 mL (1.62 mL – 1.44 mL = 0.18 mL). The results of the difference in fluid volumes after the two stages were summed up and amounted to 0.56 mL (0.38 mL + 0.18 mL = 0.56 mL). Thus, two stages of degassing and dust extraction reduced the liquid volume by 0.56 mL (2.0 mL – 1.44 mL = 0.56 mL) and in parallel increased its AC_A_ by 0.56 mL, or 28 %. Consequently, the AC_A_ of OGB granular fraction (Cerabone) because of degassing was 0.56 mL and increased by 28 % from the initial adsorption capacity. The scheme of the two-stage preparation of the OGB (passive and active degassing with the removal of gas bubbles, coarse and fine particle matters from the surface and pores of the granular fraction) and the corresponding calculations using the example of Cerabon® are shown in Fig. 4.

Similarly, the AC_A_ calculation extended to other OGBs: Maxresorb, Bio-OSS, Xenograft Collagen, Osteon Ⅱ, MSP (Table 5).

**Table 5:** Indicators of changes in the physical property of the OGBs (M±SD).

OGB	W_mА_, %	W_mB_, %	АC_A_, mL	АC_T_, mL
Xenograft Collagen	129.27 ± 0.67	87.47 ± 0.83	0.997 ± 0.015	0.895 ± 0.015
Osteon II	93.63 ± 1.06	31.73 ± 1.40	0803 ± 0.015	0.803 ± 0.015
Maxresorb	115.10 ± 1.65	41.93 ± 0.47	0.753 ± 0.015	0.753 ± 0.015
MSP	80.12 ± 0.83	28.60 ± 0.56	0.681 ± 0.015	0.681 ± 0.015
Cerabone	59.13 ± 0.70	19.10 ± 0.70	0.563 ± 0.015	0.563 ± 0.015
Bio-OSS	131.13 ± 1.29	59.00 ± 0.40	0.703 ± 0.015	0.386 ± 0.015

#### 3.2.2 True adsorption capacity indicator of the OGB

The determination of the OGB's AC_T_ incorporated AC_A_, W_mA_, and W_mB_ indicators (Section 2.3), along with micropore volume, macropore presence, and through pores in the granules (Table 5). Study determined that the nature of the distribution of macropores and micropores in OGB granules determines the method of calculating AC_T_:

• For OGBs such as Maxresorb, Cerabone, Osteon Ⅱ, and MSP, AC_T_ corresponds to AC_A_ (Table 5), since the micropores’ volume is < 1 cm^3^/g and the presence of macropores, through pores and channels in the granules is determined;
• For OGB such as Xenograft Collagen, АC_T_ = АC_A_ × ((W_mА_ – W_mB_) + W_mB_ × 0,85) / W_mА_, since the volume of micropores is in the range of 1 cm^3^/g to 10 cm^3^/g, and the presence of macropores, through pores and channels in the granules is determined. 0.85 correction factor that takes into account only macropores in W_mB_;
• For OGB such as Bio-OSS, АC_T_ = АC_A_ × (W_mА_ – W_mB_) / W_mА_, since the volume of micropores is > 10 cm^3^/g and single macropores are determined in sight.

Using AC_A_, micropore volume, W_mA_, and W_mB_, researchers can produce OGB with a given true adsorption capacity of the OGB granular fraction. The larger the volume of micropores in OGB, the lower the AC_T_ index and the adsorption property of OGB. The adsorption property of the OGB mostly depend on the number of macropores in 1 cm of the granular fraction.

The study also found that the AC_T_ value correlates with the number of bone growth factors (BGFs) adsorbable to OGB surfaces. To put them on 1 mL of the OGB Cerabone after preparing its external surfaces and free spaces in our manner, it is sufficient to prepare 0.56 mL of BGFs. The data show that the AC_T_ indicator of the OGB fraction corresponds to the required volume of BGFs that OGB granular fraction can adsorb. The OGB fraction’s maximal BGFs adsorption capacity (on the free granule surfaces, in the intergranular spaces, and interior pores spaces) correlates with its AC_T_. Due to air and fine particular matter on the surface, in the pores, and within the channels of the OGB granules, the AC_T_ of the OGB fraction has limitations. Hydrodynamic and mechanical shutters prevent BGFs migration and invasion of vessels and fibrous structures from the recipient site and soft tissue environment.

### 3.3 The compaction coefficient of the OGB

Study also investigated how the dynamics of changes in the physical property of the OGB fraction affect the stability of the bioengineered structure. The computation of prognostic indicators characterized the differences in the OGB fraction's physical property during the postoperative period. These indicators show the cumulative dynamics of the OGB fraction’s transformation in the scaffold after its placing in the recipient site. Table 6 contents the C_C_ of the studied OGBs.

**Table 6:** The compaction coefficient (C_C_) of the OGB (M±SD).

**OGB**	**Compaction coefficient (C**_**C**_**)**
Osteon Ⅱ	1.69 ± 0.02
Maxresorb	1.28 ± 0.01
Cerabone	1.25 ± 0.01
Xenograft Collagen	1.24 ± 0.01
MSP	1.03 ± 0.01
Bio-OSS	1.02 ± 0.02

The preparation of the OGB granular fraction using our method increased both the surface area and the volume of the OGB free spaces that were available for the BGFs adsorption. When addressing a bone defect of the jaws using the method of guided bone regeneration, the required volume of the OGB fraction must be determined considering the C_C_ parameter. The C_C_ of the OGB granular fraction is a prognostic indicator that collectively reflects the dynamics of changes in the physical property of the scaffold in the postoperative period. Using C_C_ of the OGB when planning the addressing of jawbone defect allows 3D modeling of the scaffold relief, considering the biotransformation of the OGB and maintains the planned volume of the scaffold, reducing the risk of uncontrolled changes in its volume in the recipient site.

### 3.4 Scanning electron microscopy of the OGBs granular fractions

**Fig. 5:**
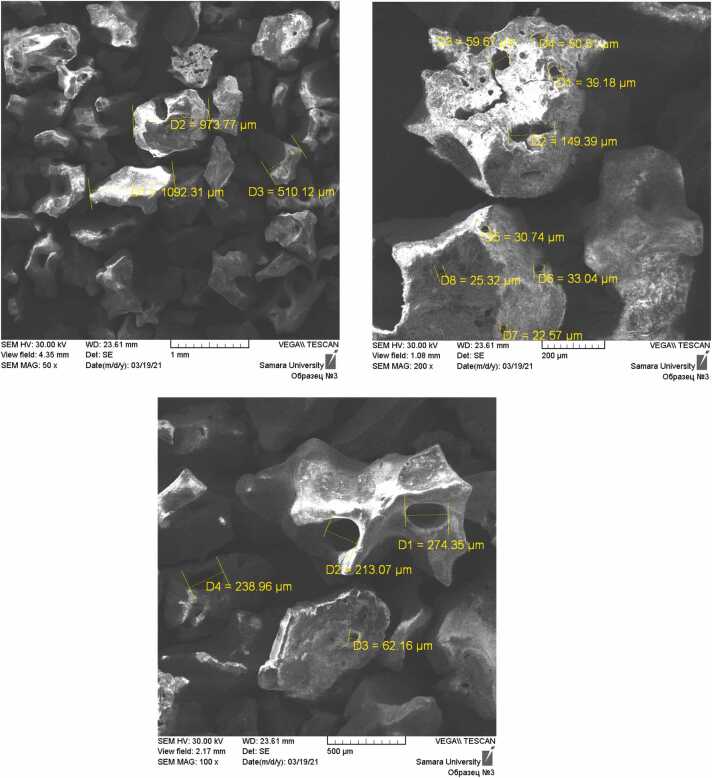
SEM micrographs of Cerabone OGB with particles and pores measuring.

SEM displays the distributions of particle sizes. The latter that differ from the manufacturers’ specifications can greatly influence on porosity. Consequently, Cerabone and Xenograft Collagen had a high concentration of particles smaller than 2500 μm in the samples. The distribution of macropores in their particles, which had size range from 22 up to 275 μm, makes them most porous (Fig. 5).Additionally, Maxresorb and Cerabone contained particles with a lot of porous measuring 50–190 μm. Xenograft Collagen, MSP, and Osteon II demonstrated the most uneven surfaces while the most even particle surface was in the Bio-Oss, which also had few through pores and channels.

After carrying out the preparation of OGB granular fraction according the developed protocol and studying of latter using SEM, study found that the content of course and fine particular matter decreased on the outer and inner surfaces of the channels and pores. The area of the free surfaces of pores and the mouths of through pores increased. Thus, the OGB preparation increased the adsorption capacity of the Xenograft Collagen OGB fraction by 50 %, Osteon II by 40 %, Maxresorb by 37 %, MSP by 34 %, Cerabone by 28 % and Bio-OSS by 17 %. After the second stage, the fine particulate matter and residual gases no longer remained on the inner surfaces of the OGB channels, pores, and voids.

### 3.5 Micropore volume of the OGB determined by gas adsorption

The gas adsorption method measured the specific surface area of each OGB granular sample. This stage of the research allowed us to estimate the volume of micropores in all samples of the studied OGBs. The highest value of micropores’ volume was observed in the Bio-Oss sample (88.226 cm^3^/g), followed by the xenograft collagen result (10.945 cm^3^/g). For the remaining three OGBs studied, the pore volume was significantly smaller and amounted to MSP (0.715 cm^3^/g), Cerabone (0.525 cm^3^/g), and Maxresorb (0.128 cm^3^/g) and Osteon II (0.124 cm^3^/g).

### 3.6 Fourier transform infrared spectroscopy of the OGB

The study showed that all the studied OGBs contain calcium phosphate; the samples of the Bio-Oss® and Cerabone® contain also calcium carbonate. Device detected characteristic peaks for the P-O (570–605 cm⁻¹) and O-H (3500 cm⁻¹ and 1650 cm⁻¹) bonds in all spectra of the studied samples. The spectra of the Bio-Oss®, Cerabone® and Osteon II® samples also display an additional O-H peak (3575 cm⁻¹), confirming the presence of hydroxyapatite. Furthermore, peaks responsible for the presence of C-O bonds are visible in the Bio-Oss® and Cerabone® samples (1460 cm^−1^ and 1420 cm^−1^, and 880 cm^−1^). The spectrum of the Xenograft Collagen and MSP samples also contain peaks responsible for the presence of C-H (1550 cm^−1^) and N-H (1245 cm^−1^) bonds, which indicate the presence of collagen in this sample. The surfaces of the four OGBs studied, Bio-Oss®, Maxresorb®, Cerabone® and Osteon II® has a similar chemical composition. The surface of the Xenograft Collagen and MSP samples are covered with collagen. Consequently, the preparation process does not affect the composition of the OGBs, either mineral or organic, which is crucial for their clinical application.

## 4 Discussion

For addressing the lost volume of jawbone tissue, OGB is the preferred choice. OGBs come in the form of a granular fraction, which exhibits physical property typical of bulk mixtures. This characteristic allows filling of the complex-shaped bone defects. The physical property of the OGB fraction alter after implantation in the bone defect. As a result, the volume of the implanted OGB fraction varies uncontrollably in the first three weeks after surgery [28], [29]. To address this limitation, it is necessary to establish conditions enabling controlled shape stability of the implanted fraction, based on modeling a biomimetic system with predictable OGB behavior in the recipient site. The parameters of the biomimetic porous structure include porosity, surface area, pore size, and interconnectivity [29]. For successful bone defect reconstruction, OGBs must exhibit good biocompatibility, osteoconductivity, and controlled biodegradation at all stages of reparative osteogenesis [30], [31]. Osteoconductivity reflects the ability of OGBs to provide a scaffold for new bone tissue growth within the defect area through cell adhesion, proliferation, and migration [32]. However, the biological environment of the recipient site significantly influences the biodegradation dynamics of the OGB granular fraction and the bioabsorption kinetics of BGFs [33], [34]. The host immune response may also lead to uncontrolled changes in the physicochemical properties of OGBs, resulting in postoperative complications such as deviations from the intended scaffold volume and loss of osteoconductive property [35]. To prevent such complications, it is crucial to investigate the causes of OGB granular fraction property alterations during the postoperative period. Furthermore, 3D planning of bone defect repair must account for the dynamics of these changes.

During OGB production, the particulate fraction evolves into a heterogeneous composition. As individual particles aggregate, they acquire distinct physical property characteristic of loose granular systems. A consequential byproduct of this manufacturing process is fine dust deposition - a seemingly minor yet functionally significant factor. This dust coats granule surfaces and pore openings, creating microbarriers that impede fluid dynamics while exacerbating hypoxic stress across the bioengineered construct.

Beyond mechanical obstruction, dust particles disrupt cellular communication by distorting crucial physicochemical signals. Through trans-interaction interference and heterophilic binding disruptions [36], they compromise both cell-cell dialogues and substrate recognition. Geometrically, dust deposition alters pore architecture, transforming potential interconnected channels into fragmented cavities. The consequent pore mouth occlusion prevents the formation of an integrated drainage system, while trapped air pockets further obstruct these microconduits.

This dual phenomenon of dust accumulation and air entrapment precipitates a cascade of functional declines [37]:

• Reduction in effective surface area accessible for molecular interactions;
• Impaired adsorption capacity across the entire scaffold;
• Restricted mobility of bioactive molecules and complexes into the implant's core.

The physical metamorphosis of OGB continues when prepared ex tempore and exposed to the recipient site's biological milieu. Contemporary studies reveal that homogenization patterns and interphase boundary evolution between particulate subtypes depend not merely on system kinetics, but fundamentally on the initial spatial configuration of granular elements [1], [5]. Investigation of these processes is justified by the need to develop appropriate models for in vitro simulation of OGB fraction behavior comparable to in vivo processes. Importantly, such modeling should accurately reflect the behavior of the entire granular OGB fraction within the recipient site. These models should be cost-effective and user-friendly.

A standardized protocol for OGB testing and clinical implementation needs to be established. For this purpose, granular limestone fractions can serve as a valid model material. Limestone - a sedimentary rock primarily of organic (biogenic) origin – consists of calcite or calcareous skeletal remains of biological organisms [38]. The calcium carbonate component of limestone undergoes gradual aqueous dissolution, decomposing into carbon dioxide and the corresponding basic oxide [39].

Key physical features of shell limestone include:

• Bulk density: ∼800 kg/m³
• Compressive strength: 0.4 MPa
• Water absorption: 0.1–2.1 %
• Porosity: 0.5–35 %

These parameters reflect fundamental physical characteristics that are comparable to those of OGB fractions.

Using such parameters as АC_A_, micropore volume, W_mА_, and W_mB_, researches can manufacture OGB with a set adsorption capacity according to the existing clinical requirements. The larger the volume of micropores in OGB, the lower the AC_T_ indicator and the lower the adsorption property of OGB granular fraction. The latter parameter predominantly depend on the quantity of macropores in 1 cm of the OGB fraction.

Our study found that the Cerabone, Osteon II, Maxresorb, and MSP OGBs have a volume of micropores of < 1 cm^3^/g, so the W_mB_ parameter reflected only macropores. At the same time, W_mА_ is proportionate to АC_A_. Thus, АC_T_ of Cerabone, Osteon II, Maxresorb, and MSP OGB fractions correspond to their АC_A_ (Table 5). The volume of micropores in the Xenograft Collagen OGBs ranges between 1 сm^3^/g and 10 cm^3^/g with АC_T_ being different from АC_A_. In general, АC_T_ depends on W_mB_, with W_mB,_ in turn, being represented mainly by macropores and partly by micropores. The calculation of AC_T_ for the Xenograft Collagen OGB incorporated a correction coefficient to subtract micropore volume from the W_mB_ value. The Bio-Oss demonstrates the highest microporous volume among comparable OGBs. SEM analysis reveals that Bio-Oss OGB granules exhibit a uniform surface morphology with only occasional macropores visible in the field of view. Consequently, when determining the АC_T_ index for the Bio-Oss OGB granular fraction, the calculations excluded the W_mB_ parameter.

The presence of macropores is necessary to improve reparative osteogenesis and increase the surface area of the OGB granular fraction [40], [41], [42], [43]. Large pores enhance oxygen diffusion into the internal parts of the fraction, thus increasing the viability of cells [40]. Researchers describe the 38.3 μm bimodal pore distribution improves cell seeding efficiency while maintaining viability and promoting proliferation [44].

Recent studies highlight the remarkable capacity of micropores to enhance transplant functionality. These microscopic voids facilitate the adsorption of cellular components, mediate tissue diffusion dynamics, and promote vascular ingrowth. By improving ion exchange with host tissues, micropores critically support cell adhesion, proliferation, and differentiation [45], [46], [47], [48]. Notably, they generate capillary forces that actively draw biological fluids and cells toward scaffold surfaces, thereby amplifying regenerative potential in vivo [49], [50].

However, this microporous advantage comes with paradoxical limitations. Evidence demonstrates that micropores can paradoxically hinder angiogenesis and disrupt the migration/differentiation of osteoblast precursors [51]. This stems from a fundamental size discrepancy: while osteoblasts (20 µm) and osteoclasts (up to 180 µm) navigate the extracellular environment, micropores (≤0.03 µm) become physical bottlenecks. The resulting cellular congestion leads to pore occlusion [52], creating a biological traffic jam that compromises scaffold functionality.

Dust contamination presents another critical challenge, significantly suppressing surface proliferation activity [48]. This underscores the vital importance of preserving macropores and interconnected channels within the granular matrix. These larger voids serve dual purposes:

• Accommodating additional OGB volume to enhance structural stability at the implantation site;
• Maintaining fluidic highways for cellular trafficking and molecular exchange.

Our optimized OGB preparation method achieves unprecedented cleanliness through a phased degassing approach:

### 4.1 Passive degassing phase

• Removes coarse particulate matter from external surfaces
• Eliminates air pockets trapped during manufacturing

### 4.2 Active degassing phase

• Employs ultrasonic cavitation to generate targeted hydraulic shocks
• Exploits surface roughness-induced wave interference to dislodge fine particle matter
• Liberates submicron debris from internal pore networks

This process transforms the scaffold’s microarchitecture. Collapsing microbubbles coalesce and evacuate through fluid channels [53], [54] and purified pore networks exhibit enhanced surface area for microcirculatory interaction, molecular complex adsorption and cellular migration from implantation margins. The resultant scaffold demonstrates superior biointegration, with interconnected pores serving as conduits for interstitial fluid perfusion, migration pathways for progenitor cells and deposition templates for extracellular matrix.

## 5 Conclusions

In guided bone regeneration procedures for jawbone defects, precise calculation of the OGB granular fraction volume is paramount. This calculation must incorporate the compression coefficient (C_C_) of the OGB scaffold system. The C_C_ serves as predictive metric, allowing clinicians to anticipate postoperative changes in the OGB’s physical characteristics. By integrating C_C_ during the preoperative 3D planning phase, surgeons can design constructs that account for expected OGB transformations, maintain the intended scaffold volume post-implantation and minimize risks of uncontrolled volumetric changes at the recipient site.

Our findings demonstrate that the BGFs required volume directly correlates with the OGB’s AC_T_. This parameter quantifies the maximal BGFs volume capable of interfacing with available OGB surfaces, including internal pore linings, external particle surfaces and intergranular spaces. However, AC_T_ is compromised by air entrapment within the porous architecture, fine particle matter contamination on particle surfaces and pore networks and hydrodynamic/mechanical traps in deep pores that impede BGFs integration, vascular infiltration and fibrous tissue migration.

Through controlled manufacturing, researches can tailor OGB adsorption capacity by modulating key parameters such as AC_A_, micropore volume distribution, W_mA_/W_mB_. Notably, while increased micropore volume reduces AC_T_; it enhances interstitial fluid diffusion, cell adhesion/migration dynamics and neoangiogenesis potential. This enhancement stems from an optimized free surface area-to-volume ratio across external and internal OGB structures. Refined manufacturing protocols and ex tempore clinical preparation techniques can further improve adsorption and drainage properties.

## Research Data Availability

Photo materials are available at the link: https://zenodo.org/api/records/15254920/draft/files/Downloads.zip/content

## Funding

This research received no external funding.

## CRediT authorship contribution statement

**Denis G. Alekseev:** Writing – review & editing, Conceptualization. **Oleg V. Slesarev:** Writing – original draft, Project administration. **Darya V. Malchikova:** Writing – original draft, Visualization, Data curation. **Vyacheslav G. Belanov:** Investigation, Data curation. **Vladimir I. Platonov:** Validation, Investigation. **Natalia A. Maksimenko:** Methodology, Data curation. **Ravshanbek B. Abdullaev:** Supervision, Formal analysis.

## Declaration of Generative AI and AI-assisted technologies in the writing process

The authors of the manuscript confirm that there is no plagiarism of text and graphic objects in the paper and that AI-assisted technologies were not involved in the preparation of the material.

## Conflict of Interest

The authors declare no conflict of interest.

## References

[1] Zhang L., Yang G., Johnson B.N., Jia X. (2019 Jan 15). Three-dimensional (3D) printed scaffold and material selection for bone repair. Acta Biomater.

[2] Fernandez de Grado G., Keller L., Idoux-Gillet Y., Wagner Q., Musset A.M., Benkirane-Jessel N. (2018). Bone substitutes: a review of their characteristics, clinical use, and perspectives for large bone defects management. J Tissue Eng.

[3] Hsu E.L., Stock S.R. (2020). Growth factors, carrier materials, and bone repair. Handb Exp Pharm.

[4] Ferlin K.M., Prendergast M.E., Miller M.L., Kaplan D.S., Fisher J.P. (2016 Mar 1). Influence of 3D printed porous architecture on mesenchymal stem cell enrichment and differentiation. Acta Biomater.

[5] Lutzweiler G., Ndreu Halili A., Engin Vrana N. (2020 Jun 29). The overview of porous, bioactive scaffolds as instructive biomaterials for tissue regeneration and their clinical translation. Pharmaceutics.

[6] Ali D., Sen S. (2018). Computational fluid dynamics study of the effects of surface roughness on permeability and fluid flow-induced wall shear stress in Scaffolds. Ann Biomed Eng.

[7] Callens S.J.P., Uyttendaele R.J.C., Fratila-Apachitei L.E., Zadpoor A.A. (2020 Feb). Substrate curvature as a cue to guide spatiotemporal cell and tissue organization. Biomaterials.

[8] Kasten P., Beyen I., Niemeyer P. (2008). Porosity and pore size of beta–tricalcium phosphate scaffold can influence protein production and osteogenic differentiation of human mesenchymal stem cells: an in vitro and in vivo study. Acta Biomater.

[9] Margarida Figueiredo, Jose Henriques, Gabriela Martins, Fernando Guerra, Fernando Judas, Helena Figueiredo. Physicochemical Characterization of Biomaterials Commonly Used in Dentistry as Bone Substitutes—Comparison with Human Bone. Journal of Biomedical Materials Research Part B: Applied Biomaterials. Published online 10 November 2009 in Wiley InterScience (〈www.interscience.wiley.com〉). DOI: 10.1002;jbm.b.31529. 10.1002/jbm.b.31529 19904820

[10] Karageorgiou V., Kaplan D. Porosity of 3D biomaterial scaffolds and osteogenesis. Journal of Biomaterials. Published online 7 April 2005 in ScienceDirect (〈www.sciencedirect.com〉). DOI: 10.1016/j.biomaterials.2005.02.002. 10.1016/j.biomaterials.2005.02.002 15860204

[11] Trajkovski Branko, Jaunich Matthias, Müller Wolf-Dieter, Beuer Florian, Zafiropoulos Gregory-George, Houshmand Alireza (2018). Hydrophilicity, viscoelastic, and physicochemical properties variations in dental bone. Materials.

[12] Zhou W.N., Pan Y.C., Tang Y.C., Hou W., Wu D.M., Yuan H. (2019). Comparative outcomes of block and cancellous iliac bone grafting in older unilateral alveolar cleft patients. Cleft Palate Craniofac J.

[13] Aloy-Prosper A., Maestre-Ferrin L., Penarrocha-Oltra D., Penarrocha-Diago M. (2011). Bone regeneration using particulate grafts: an update. Med Oral Patol Oral Cir Bucal.

[14] Troeltzsch M., Troeltzsch M., Kauffmann P., Gruber R., Brockmeyer P., Moser N. (2016). Clinical efficacy of grafting materials in alveolar ridge augmentation: a systematic review. J Craniomaxillofac Surg.

[15] Lee S.H., Choi B.H., Li J., Jeong S.M., Kim H.S., Ko C.Y. (2007). Comparison of corticocancellous block and particulate bone grafts in maxillary sinus floor augmentation for bone healing around dental implants. Oral Surg Oral Med Oral Pathol Oral Radio Endod.

[16] Alias Mohd Almie, Buenzli Pascal R. (January 10, 2017). Modeling the effect of curvature on the collective behavior of cells growing new tissue. Biophys J.

[17] Hegarty-Cremer S.G.D., Simpson M.J., Andersen T.L., Buenzli P.R. (2021 Jul 7). Modelling cell guidance and curvature control in evolving biological tissues. J Theor Biol.

[18] Stuckensen K., Schwab A., Knauer M., Muin ~os-Lo ´pez E., Ehlicke F., Reboredo J. (2018). Tissue mimicry in morphology and composition promotes hierarchical matrix remodeling of invading stem cells in osteochondral and meniscus Scaffolds. Adv Mater.

[19] Abdal Dayem A., Lee S., Choi Y., H, Cho S.-G. (2018). The impact of adhesion molecules on the *in vitro* culture and differentiation of stem cells. Biotechnol J.

[20] Pina S., Ribeiro V.P., Marques C.F., Maia F.R., Silva T.H., Reis R.L. (2019). Scaffolding strategies for tissue engineering and regenerative medicine applications. Mater (Basel Switz).

[21] Ali D., Sen S. (2018). Computational fluid dynamics study of the effects of surface roughness on permeability and fluid flow-induced wall shear stress in Scaffolds. Ann Biomed Eng.

[22] Zhu Guanyin, Zhang Tianxu, Chen Miao, Yao Ke, Huang Xinqi, Zhang Bo, Li Yazhen, Liu Jun, Wang Yunbing, Zhao Zhihe (November 2021). Bone physiological microenvironment and healing mechanism: Basis for future bone-tissue engineering scaffolds. Bioact Mater.

[23] Sun Y., Oshinowo O., Myers D.R., Lam W.A., Alexeev A. (2021 Dec 27). Resolving the missing link between single platelet force and clot contractile force. iScience.

[24] Maia F.R., Bastos A.R., Oliveira J.M., Correlo V.M., Reis R.L. (2022). Recent approaches towards bone tissue engineering. Bone.

[25] Slesarev O.V., Bairikov I.M., Malchikova D.V., Platonov V.I., Jordanishvili A.K., Muzykin M.I., Gribkova O.V., Komarova M.V., 2758570 C1, 2021.

[26] Higashi T., Okamoto H. (1995). The effect of ultrasonic irrigation before and after citric acid treatment on collagen fibril exposure: an in vitro SEM study. J Periodo.

[27] Shetty B., Dinesh A., Seshan H. (2008). Comparitive effects of tetracyclines and citric acid on dentin root surface of periodontally involved human teeth: a scanning electron microscope study. J Indian Soc Periodo.

[28] Jemt T., Lekholm U. (2003). Measurements of buccal tissue volumes at single-implant restorations after local bone grafting in maxillas: a 3-year clinical prospective study case series. Clin Implant Dent Relat Res.

[29] Araújo M., Sonohara M., Hayacibara R., Cardaropoli G., Lindhe J. (2002 Dec). Lateral ridge augmentation by the use of grafts comprised of autologous bone or a biomaterial. An experiment in the dog. Clin Periodo.

[30] Chen X., Fan H., Deng X., Wu L., Yi T., Gu L., Zhou C., Fan Y., Zhang X. (2018). Scaffold structural microenvironmental cues to guide tissue regeneration in bone tissue applications. Nanomaterials.

[31] Haugen H.J., Lyngstadaas S.P., Rossi F., Perale G. (2019). Bone grafts: which is the ideal biomaterial. J Clin Periodo.

[32] Yamada M., Egusa H. (2018). Current bone substitutes for implant dentistry. J Prosthodont Res.

[33] Navarro M., Michiardi A., Castaño O., Planell J.A. (2008). Biomaterials in orthopaedics. J R Soc Interface.

[34] Schorn L., Sine A., Berr K., Handschel J., Depprich R., Kübler N.R., Sproll C., Rana M., Lommen J. (2022). Influence of xenogeneic and alloplastic carriers for bone augmentation on human unrestricted somatic stem cells. Materials.

[35] Luo Z., Hu Y., Wang Q. (1997). The experimental studies of immune response of antigen-extracted bovine cancellous bone grafting. Zhonghua Wai Ke Za Zhi.

[36] Abdal Dayem A., Lee S., Choi Y., H, Cho S.-G. (2018). The impact of adhesion molecules on the *in vitro* culture and differentiation of stem cells. Biotechnol J.

[37] Burger E.H., Klein-Nulend J. (1999). Mechanotransduction in bone—role of the lacuno-canalicular network. FASEB J.

[38] Baino F. (2011). Biomaterials and implants for orbital floor repair. Acta Biomater.

[39] Shixaliyev K.S. (2018). The study of the properties of limestone grains surfaces and determination of optimal proportions between breakstone and limestone. Brit J Innov Sci Technol.

[40] Massalimov I.A., Massalimov B.I., Akhmetshin B.S., Urakaev F.Kh, Burkitbaev M.M. (2020). Transformation of limestone-shell rock mining waste by impregnation with polysulfide solutions. Nanotechnol Constr A Sci InternetJ.

[41] Bertazzo S., Zambuzzi W.F., Campos D.D., Ogeda T.L., Ferreira C.V., Bertran C.A. (2010). Hydroxyapatite surface solubility and effect on cell adhesion. Colloids Surf B Biointerfaces.

[42] Cyster L.A., Grant D.M., Howdle S.M., Rose F.R., Irvine D.J., Freeman D., Scotchford C.A., Shakesheff K.M. (2005). The influence of dispersant concentration on the pore morphology of hydroxyapatite ceramics for bone tissue engineering. Biomaterials.

[43] Tian T., Liao J., Zhou T., Lin S., Zhang T., Shi S.R., Cai X., Lin Y. (2017). Fabrication of calcium phosphate microflowers and their extended application in bone regeneration. ACS Appl Mater Interfaces.

[44] Zambuzzi W.F., Oliveira R.C., Pereira F.L., Cestari T.M., Taga R., Granjeiro J.M. (2006). Rat subcutaneous tissue response to macrogranular porous anorganic bovine bone graft. Braz Dent J.

[45] Salerno A., Guarnieri D., Iannone M., Zeppetelli S., Netti P.A. (2010). Effect of micro- and macroporosity of bone tissue three-dimensional-poly(epsilon-caprolactone) scaffold on human mesenchymal stem cells invasion, proliferation, and differentiation in vitro. Tissue Eng Part A.

[46] Annaz B., Hing K.A., Kayser M., Buckland T., Di S.L. (2004). An ultrastructural study of cellular response to variation in porosity in phase-pure hydroxyapatite. J Microsc.

[47] da Cruz A.C., Pochapski M.T., Daher J.B., da Silva J.C., Pilatti G.L., Santos F.A. (2006). Physico-chemical characterization and biocompatibility evaluation of hydroxyapatites. J Oral Sci.

[48] Hing K.A., Annaz B., Saeed S., Revell P.A., Buckland T. (2005). Microporosity enhances bioactivity of synthetic bone graft substitutes. J Mater Sci Mater Med.

[49] Murugan R.S., Panduranga R.K. (2006). Nanoporous hydroxy-carbonate apatite scaffold made of natural bone. Mater Lett.

[50] Turco G., Porrelli D., Marsich E., Vecchies F., Lombardi T., Stacchi C., Di Lenarda R. (2018). Three-dimensional bone substitutes for oral and maxillofacial surgery: biological and structural characterization. J Funct Biomater.

[51] Polak S.J., Rustom L.E., Genin G.M., Talcott M., Wagoner Johnson A.J. (2013). A mechanism for effective cell-seeding in rigid, microporous substrates. Acta Biomater.

[52] Rustom L.E., Boudou T., Lou S., Pignot-Paintrand I., Nemke B.W., Lu Y., Markel M.D., Picart C., Wagoner Johnson A.J. (2016). Micropore-induced capillarity enhances bone distribution in vivo in biphasic calcium phosphate scaffolds. Acta Biomater.

[53] Bobbert F.S.L., Zadpoor A.A. (2017). Effects of bone substitute architecture and surface properties on cell response, angiogenesis, and structure of new bone. J Mater Chem B.

[54] GI, E. Cavitation mechanism of ultrasonic melt degassing. Ultrasonics Sonochemistry 1995, 2(2), 137-141, doi:10.1016/1350-4177(95)00020-7.

